# Efficient and Selective Removal of Organic Cationic Dyes by Peel of Brassica juncea Coss. var. gemmifera Lee et Lin-Based Biochar

**DOI:** 10.3390/molecules28083353

**Published:** 2023-04-11

**Authors:** Tao-Tao Shi, Xin-Yu Jiang, Jin-Gang Yu

**Affiliations:** College of Chemistry and Chemical Engineering, Central South University, Changsha 410083, China

**Keywords:** Brassica juncea var. gemmifera Lee et Lin, organic dyes, bio-adsorbent, adsorption selectivity, vacuum pyrolysis

## Abstract

The design and preparation of cheaper, greener and more efficient adsorbents is essential for the removal of pollutants by adsorption. In this study, biochar was prepared from peel of Brassica juncea var. gemmifera Lee et Lin (PoBJ) using a facile, low-temperature and vacuum pyrolysis, and the adsorption mechanism toward organic dyes in aqueous solution was elucidated. The adsorbent was characterized by XPS, FT-IR and SEM, and zeta potential techniques. The adsorption ability of PoBJ biochar for cationic dyes (methylene blue, brilliant green, calcein-safranine, azure I, rhodamine B), anionic dyes (alizarin yellow R), and neutral dyes (neutral red) revealed that the biochar exhibited adsorption selectivity toward cationic dyes. The effects of different factors on the adsorption performance of PoBJ biochar, as well as the adsorption kinetics and thermodynamics, were further investigated by using methylene blue as the model adsorbate. These factors included temperature, pH, contact time and dye concentration. The experimental results showed that BJ280 and BJ160 (prepared at 280 °C and 160 °C, respectively) possessed relatively higher adsorption capacity of 192.8 and 167.40 mg g^−1^ for methylene blue (MB), respectively, demonstrating the possibility of utilization of PoBJ biochar as a superior bio-adsorbent. The experimental data of BJ160 toward MB were correlated with various kinetic and isothermal models. The results indicated that the adsorption process was consistent with the Langmuir isotherm model and nonlinear pseudo-second-order kinetic model. Thermodynamic parameters indicated that the adsorption of MB onto BJ160 was exothermic. Thus, the low-temperature prepared PoBJ biochar was an environmentally friendly, economic and efficient cationic dye adsorbent.

## 1. Introduction

Organic dyes play an important role in various industries such as textile, leather, paint, food, cosmetic and paper-making [1]. To meet the increasing industrial demand, it is estimated that 1.6 million tons of dyes are produced annually, of which 10–15% are discarded into the environment as wastewater [2]. The presence of organic dyes in water, even at very low concentrations, can abate the passage of sunlight and reduce the levels of dissolved oxygen. The inhibited photosynthesis reduces the overall oxygen content, which will cause harmful and irreversible effects to the lives of aquatic species [3]. Methylene blue (MB) is one of the most commonly used substances for dyeing cotton, wood and silk. MB would be released into the environment with industrial waste water. MB is toxic, carcinogenic and non-biodegradable, and it is dangerous to human health and the environment. MB will be chronically present in the aquatic environment, and therefore water will be highly contaminated. Tetraplegia, jaundice, shock, tissue necrosis, vomiting, rapid heart rate and cyanosis are all symptoms of intense exposure to MB [4]. Therefore, the removal of residual toxic dyes from wastewater is essential to reduce the threat to the ecosystem.

Dyes are highly soluble in water, which makes it difficult to remove them by conventional methods [5]. Common strategies for dyes removal include physical, chemical, and biological treatments. Among them, adsorption has been recognized as an efficient and environmentally friendly method due to its simplicity of operation at ambient temperature and pressure, as well as low cost [6]. The practical implementation of adsorption technology greatly depends on the suitable selection and utilization of efficient adsorbents. The research and development of cheap, low- or non-toxic and recyclable adsorbents is a major concern for adsorption technology.

Biochar, an amorphous carbon material with high carbon content, could be obtained by thermochemical pyrolysis of biomass at temperatures below 700 °C under oxygen-limited conditions [7]. Due to its outstanding physicochemical properties, such as high specific surface area, high porosity, and abundant surface functional groups, biochar has shown great potential for various applications, including carbon sequestration, bioenergy production, energy storage, soil amendment, biomass waste management, and water purification [8,9]. Biochar is a renewable resource with good economic and environmental benefits, and it is also an ideal resource for the treatment of water pollutants in environmental technologies [10,11]. Previous studies have reported the removal of organic dyes from wastewater using biochar produced from different biomass, such as algae and plant biomass, forest, and domestic residues, animal waste, sewage sludge, etc. Gasification, pyrolysis, torrefaction and hydrothermal carbonization have been utilized for thermal decomposition of biomass. In addition, microwave-assisted pyrolysis and hydrothermal carbonization have also been utilized for faster production of biochar [12,13]. Biochar materials derived from sludge-rice husk [14], pigments-extracted macroalgae [15], litchi peel [16], wakame (Undaria pinnatifida) [15], etc., could be used for highly efficient removal of organic dyes from contaminated wastewaters. However, the selective adsorption and separation of organic dyes could be achieved by rationally developed adsorbents with specific recognitive sites [17,18,19]. Organic dyes could be divided into cationic, neutral and anionic pigments. To selective adsorption organic dyes, the preparation of biochar-based composites has been widely adopted [20,21,22]. However, the disadvantages of these biochar-based adsorbents, such as being complicated, time-consuming and having a laborious production procedure, as well as uneconomical properties are obvious. To facilely and rationally construct novel biochar-based adsorbents with excellent selectivity, the thermal treatment conditions/methods and the kind of biomass undoubtedly need to be carefully considered.

Brassica juncea var. gemmifera Lee et Lin (BJ), a popular vegetable that is grown in southwest China, is widely consumed and extremely popular due to its nutritious qualities, as well as its mustard aroma and good taste. However, the peel of BJ (PoBJ) is thick and inedible. A high weight ratio of PoBJ, that is approximately 15% of the total weight of BJ, can be detected. The discard of PoBJ would cause a waste of resources and even negative environmental impacts. PoBJ possesses a large amount of oxygen (O)-, sulfur (S)- and nitrogen (N)-containing functional groups such as aromatic thioglucosides, cellulose and lignin [23], which could interact with other molecules through hydrogen bonding, providing a potential for adsorption of organic contaminants in aqueous solutions.

In this study, PoBJ biochar was prepared by vacuum pyrolysis of PoBJ under relatively lower temperatures to achieve energy conservation and pollution reduction. In addition, the exhausted gas was well absorbed by an absorption tank. The adsorption experiments exhibited that PoBJ biochar possessed high adsorption selectivity toward cationic dyes. In order to investigate the adsorption mechanism, the compositions and microstructure of PoBJ biochar were explored using X-ray photoelectron spectroscopy (XPS), Fourier transform infrared spectroscopy (FT-IR) and scanning electron microscopy (SEM), and zeta potential analysis. By using methylene blue (MB) as the model adsorbate, batch adsorption experiments were conducted to explore the adsorption kinetics and thermodynamics, as well as the reusability and adsorption efficiency of PoBJ biochar.

## 2. Results and Discussions

### 2.1. SEM Analysis

**Figure 1:**
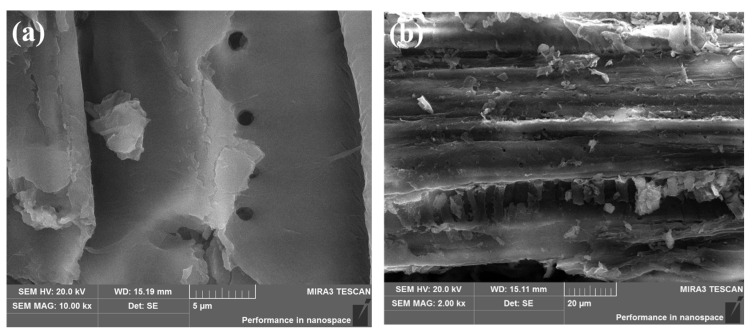
SEM images of BJ160: (**a**) Magnification: 10,000×; (**b**) Magnification: 2000×.

SEM analysis was used to study the surface morphological changes of PoBJ biochar (Figure 1). Obviously, BJ160 possesses sheet-like structures. The irregular, rough, homogeneous structures with a few pores unevenly distributed on the surface could be observed, which facilitated the fast migration of dye molecules to the biochar.

### 2.2. FT-IR Analysis

**Figure 2:**
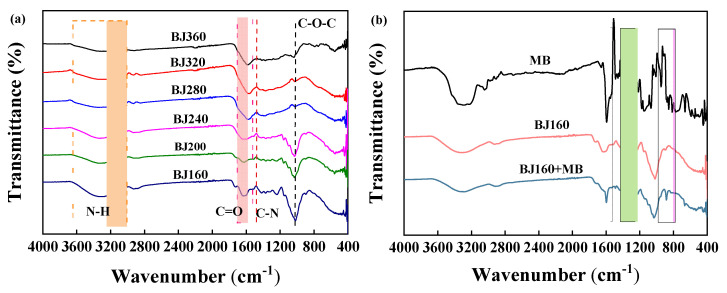
FT-IR spectra of PoBJ biochar: (**a**) BJ prepared at different pyrolysis temperatures; (**b**) MB, and BJ160 pre- and post-adsorption of MB.

The FT-IR spectra of PoBJ materials pre- and post-adsorption of MB were recorded (Figure 2). It is obvious that PoBJ biochars are rich in oxygen-containing functional groups. The functional groups of PoBJ biochar pyrolyzed at different temperatures were almost identical (Figure 2a). The broad peak at around 3333 cm^−1^ is associated with the stretching vibration of the N-H bond, and the peak at around 1600 cm^−1^ corresponds to the stretching vibration of the -C=O- bond [24,25]. The peak at 1480 cm^−1^ could be attributed to the stretching vibration of the -C-N bond [26,27]. The peak at 1021 cm^−1^ could be assigned to the stretching vibration of the C-O-C bond [28]. As the pyrolysis temperature increases, the thermal cleavage of the C-O and C-N bonds occurs under anaerobic conditions, generating volatile substances which would be easily absorbed and purged by a gas absorption tank. Other functional groups such as hydroxyl (-OH) and C-O-C groups would be also gradually cleaved, resulting in decreased peak height [29]. The FT-IR spectra of MB and BJ160 pre- and post-adsorption of MB were recorded (Figure 2b). In comparison with pristine BJ160, BJ160 post-adsorption of MB exhibit distinct changes in peak width and intensity at 1600 cm^−1^, 1400 cm^−1^ and 850 cm^−1^, confirming the successful adsorption of MB by the adsorbent.

### 2.3. XPS Analysis

**Figure 3:**
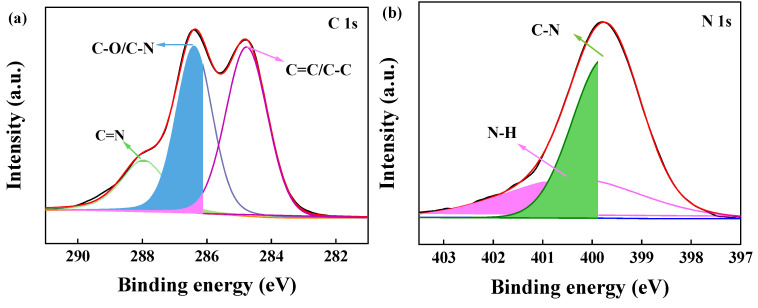

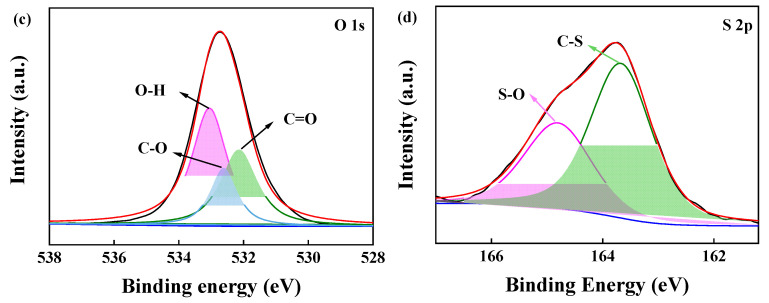
XPS-peak-differentiation-imitating analyses of BJ160: (**a**) C1s (green: C=N; blue: C-O/C-N; pink: C=C/C-C), (**b**) N1s (green: C_N; pink: N-H), (**c**) O1s (green: C=O; blue: C-O; pink: O-H) and (**d**) S2p (green: C-S; pink: S-O).

To further investigate the chemical compositions of the adsorbent, BJ160 was analyzed by XPS, and the XPS-peak-differentiation-imitating analyses of C1, N1, O1, and S1 spectra of BJ160 are shown (Figure 3). The peak at 287.96 eV (C1s) represents the C=N bond [30], and the peak at 286.38 eV (C1s) is associated with the C-O and C-N bonds [31], while the peak at 284.76 eV corresponds to the C=C and C-C peaks (Figure 3a) [32]. For the N1s peaks, the peak corresponding to the C-N bond is located at 399.72 eV [33], and the peak corresponding to N-H is located at 400.40 eV (Figure 3b). For the O1s peaks, the peak at 532.15 eV corresponds to the C=O double bond of carbonyl group [34], and the peak at 532.62 eV corresponds to the C=O double bond, while the peak at 533.05 eV corresponds to the O-H bond of carboxyl group (Figure 3c) [35]. For the S2p peaks, the peak at 164.83 eV corresponds to S-O bond, and the peak at 163.67 eV corresponds to C-S bond. The XPS results demonstrated the presence of O-, N- and S-containing functional groups on the surface of BJ160.

### 2.4. Adsorption Performances

**Figure 4:**
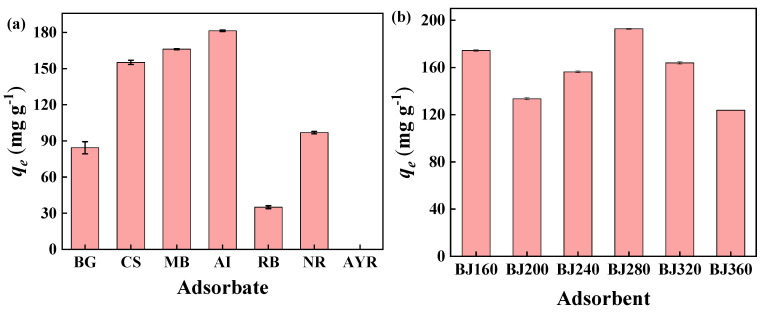
Adsorption performances of PoBJ biochar: (**a**) adsorption capacities of BJ160 toward different dyes in aqueous solutions (dye concentration = 50 mg L^−1^, adsorbent dosage = 5.0 mg, and contact time = 60 min, *T* = 25 °C); (**b**) adsorption of MB by various BCs (BJ160, BJ200, BJ240, BJ280, BJ320 and BJ360; MB concentration = 50 mg L^−1^, adsorbent dosage = 5.0 mg, and contact time = 120 min, *T* = 25 °C).

Batch adsorption experiments were carried out. The adsorption of various cationic dyes (BG, CS, MB, AI, RB), neutral dye (NR) and anionic dye (AYR) by BJ160 was investigated, and the experiments were carried out at the natural pH values of the dyestuffs (Figure 4a). The results showed that PoBJ biochar possessed relatively higher adsorption capacities toward cationic and neutral dyes, almost no adsorption occurred for anionic dye (AYR) could be observed. The obvious difference can be attributed to the O-, N- and S-containing functional groups of BJ160 were negatively charged. The results demonstrated that PoBJ biochar might be used as an adsorbent for selective adsorption of cationic dyes.

### 2.5. Effect of Pyrolysis Temperature on PoBJ Material toward MB Removal

The adsorption of MB by various PoBJ biochars (BJ160, BJ200, BJ240, BJ280, BJ320 and BJ360) was investigated (Figure 4b). In the pyrolysis temperature range 200–360 °C, the adsorption capacity of PoBJ biochar firstly increased and then decreased, and the maximum adsorption capacity of 199.72 mg g^−1^ by BJ280 could be observed. In comparison, BJ160 possessed a relatively lower adsorption capacity of 167.40 mg g^−1^ to MB. From the perspective of energy conservation and environmental protection, BJ160 was subsequently selected as the model adsorbent to explore multiple conditional parameters (such as contact temperature, contact time, initial MB concentration, solution pH value) and determine the optimal adsorption conditions.

### 2.6. Effect of Adsorption Conditions

#### 2.6.1. Effect of Contact Time

**Figure 5:**
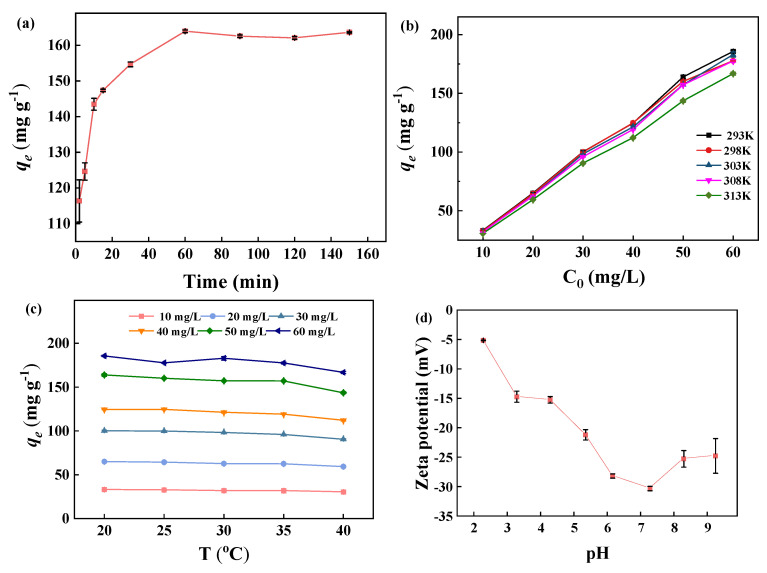

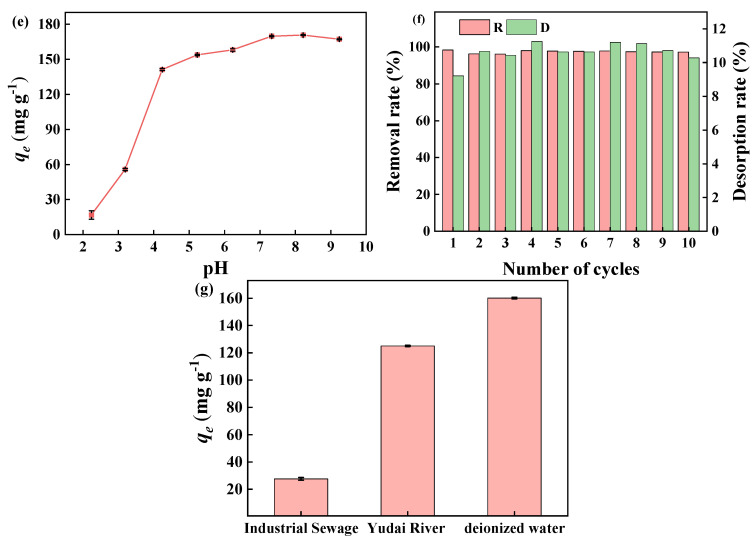
Adsorption properties of BJ160 for MB: (**a**) effect of contact time (*C*_0_ = 50.0 mg L^−1^, *T* = 25 °C); (**b**) effects of initial MB concentration (*t* = 60 min); (**c**) effect of contact temperature (*t* = 60 min); (**d**) effect of initial solution pH value (*C*_0_ = 50.0 mg L^−1^, *t* = 60 min, *T* = 25 °C); (**e**) zeta potential of BJ160 in aqueous solution at different pH values; (**f**) reusability of BJ160 for removal of MB in ten successive adsorption-desorption cycles (*C*_MB_ = 50 mg L^−1^, adsorbent dosage of 50.0 mg, and contact time of 60 min, *T* = 25 °C). (**g**) Adsorption capacity of BJ160 toward different dyes in aqueous solutions (dye concentration = 50 mg L^−1^, adsorbent dosage = 5.0 mg, *t* = 60 min, *T* = 25 °C).

The effect of contact time on the adsorption capacity of BJ160 for MB dye is shown (Figure 5a). The results showed that the adsorption capacity of 116.37 mg g^−1^ could be achieved at a contact time of 2 min. At a contact time of ≥60 min, an adsorption equilibrium could be obtained. Therefore, the adsorption process can be divided into three stages: during the first stage in the time range 0–10 min, the rapid adsorption of MB occurred, the amount of adsorption increased rapidly with an increase in contact time. Due to the abundant active sites on BJ160 and the high initial MB concentration, the diffusion rate was the highest and MB dye rapidly diffused an adsorbed onto the surface of BJ material. The second stage occurred in the time range 10–60 min, in which the rate of adsorption slowed down due to the adsorption sites of PoBJ material were gradually occupied, and MB concentration gradually decreased. During the third stage (in the time range ≥ 60 min), the adsorption of MB by PoBJ biochar basically reached an equilibrium as the contact time continuously increased, in which the MB concentration achieved a dynamic equilibrium, and the removal rate and the adsorption capacity were almost unchanged. Therefore, the subsequent experiments were performed at a contact time of 60 min.

#### 2.6.2. Effect of Initial MB Concentration and Contact Temperature

The effect of the initial concentration of MB dye on the adsorption capacity of the MB dye adsorbed by BJ160 was investigated (Figure 5b). Clearly, the adsorption capacity of BJ160 increases as the initial concentration of MB dye increases. A relatively high removal rate of 90% could be observed at an initial MB concentration of 10 mg L^−1^ and a contact temperature of 20 °C. As the initial MB concentration continuously increased, the removal rate of BJ160 for MB dye gradually decreased. At an initial MB concentration of 60 mg L^−1^, the relatively lower removal rate of 78% could be obtained. The same trends could be also observed under other temperatures, which can be explained by the amount of active sites for adsorption. The higher the initial MB concentration is, the less sufficient adsorption sites the adsorbent could provide [1]. The initial concentration of MB provides an important driving force to overcome all mass transfer resistance. This means that, with a constant amount of adsorbent, an increase in dye concentration produces a different concentration gradient on the adsorbent surface and therefore an increase in the amount of dye adsorbed per gram of adsorbent. Conversely, at higher dye concentrations, the percentage of adsorption decreases due to over-saturated active adsorption surfaces on the adsorbent [36]. Therefore, the initial dye concentration has an important influence on the adsorption efficiency of PoBJ biochar.

Contact temperature is one of the most important factors in determining the adsorption process. The contact temperature affects both the physicochemical properties of the adsorbent and the diffusion rate of the adsorbate molecules, thus determines the adsorption amount and removal rate. The effect of contact temperature on the adsorption capacity of BJ160 on MB dye is shown (Figure 5c). The change in temperature did not alter the adsorption effect of BJ160 at a constant initial MB concentration. The phenomena might be supposed to be caused by the fact that the increased migration rate of MB molecules with increasing temperature could enhance the adsorption capacity, but the dissociation constant of adsorbent-adsorbate also significantly changed [37]. Based on these combined effects, the adsorption capacity of BJ160 for MB dye remained almost unchanged with increasing temperature at a constant initial MB concentration.

#### 2.6.3. Adsorption Kinetics

The adsorption process depends on the physical and/or chemical properties of the adsorbent and the mass transfer effects. In order to reveal the mechanism of the adsorption process, the experimental data were fitted to the non-linear pseudo-first order (PFO) and the non-linear pseudo-second order (PSO) kinetic models [38,39] (Figure S1, Table S1). For the adsorption of MB by BJ160, the PSO model had the highest coefficient of determination (*R^2^*, 0.8850) [40]. Meanwhile, the calculated *q_e_* value (*q_e,cal_* = 161.7 mg g^−1^) could be well approximated by the experimental data (*q_e_*_,*exp*_ = 163.62 mg g^−1^).

#### 2.6.4. Adsorption Isotherms

To further investigate the surface properties and adsorption behaviors of MB on BJ160, two isotherm models (non-linear Langmuir and Freundlich) were used to fit the adsorption equilibrium data (Figure S2, Table S2). The higher coefficient of determination (*R^2^*) suggested that the non-linear Langmuir model was suitable to describe the adsorption of MB on BJ160, demonstrating a monolayer adsorption process on homogeneous surfaces [41]. As shown in Figure S2b, the adsorption of MB by BJ160 significantly increased at the initial stage, then gradually increased until the adsorption equilibrium was established.

#### 2.6.5. Adsorption Thermodynamics

The effect of temperature on the adsorption of MB by BJ160 was investigated by calculating the thermodynamic parameters (Figure S3). The calculated standard enthalpy (Δ*H*°, kJ mol^−1^), standard entropy (Δ*S*°, J/K/mol) and Gibbs free energy (Δ*G*°, kJ mol^−1^) are shown in Table S3 [42]. Negative Δ*H*° values could be obtained, indicating that the adsorption process is exothermic. Typically, the value of Δ*H*° can be used to reveal the nature of the adsorption process. The Δ*H*° value (−29.42 kJ mol^−1^) falls within the range of 1–40 kJ mol^−1^, indicating that physical adsorption via electrostatic attraction occurs between the adsorbent and the dye molecules at all temperatures tested [43]. The calculated entropy was negative, indicating that the adsorption reaction was a process of entropy reduction. Therefore, an increase in temperature was not beneficial to the adsorption, which was also consistent with previous discussions. As temperature increased, MB molecules might be orderly assembled on the surface of BJ160 by occupying the active sites, leading to a decrease in the degree of freedom of MB molecules at the solid-liquid interface and a decrease in entropy [44,45]. From the discussions above, it is clear that the adsorption of MB onto BJ160 is an exothermic process.

#### 2.6.6. Effect of Solution pH

It is known that the surface charge properties of an adsorbent have a great influence on its adsorption efficiency [46], so the charge properties of the adsorbent in the pH range 2–9 were investigated using zeta potential analysis (0.1 mol L^−1^ NaOH and 0.1 mol L^−1^ HCl were used to adjust solution pH values). As shown in Figure 5d, it is obvious that BJ160 was electronegative, the BJ160 particles are considered weakly anionic due to the zeta potentials are greater than −30 mV. An increasing in solution pH in the range 2–7 would produce more negative potentials. The measured zeta potential distributions of BJ160 in the solution pH range 2–9 were all electronegative, confirming its adsorption selectivity toward cationic dyes. The effect of MB solution pH in the range of 2–9 on the adsorption of MB by BJ160 was also studied (Figure 5e). The adsorption capacity of BJ160 to MB was lowest at a pH of 2, which increased significantly with an increasing pH. When the pH was in the range 7–9, the sorption of MB on BJ160 remained almost unchanged, which could be explained by the protonation and deprotonation of the active site under the action of pH. Under acidic conditions, the protonation on the surface of BJ160 occurred, producing less negative potentials on the surface of BJ160, which hindered the effective adsorption of the positively charged cationic dye. In comparison, under alkaline conditions, the deprotonation on the surface of BJ160 occurred, which consequently facilitated the intermolecular interactions with MB [47]. That is, as solution pH increased, the surface charge density increased, leading to enhanced electrostatic effects between the adsorbent and the adsorbate [48].

### 2.7. Reusability

Reusability is an important indicator for a newly developed adsorbent, which can validate the cost-effectiveness of its practical applications [49]. Briefly, 50.0 mg of BJ160 was added to 20.0 mL of MB (30 mg L^−1^) and oscillated for 60 min at 25 °C to achieve an adsorption equilibrium. Afterwards, the desorption of MB dye from BJ160 was carried out by using 40.0 mL HCl (0.1 mol L^−1^) under oscillation conditions. BJ160 was recovered by a facile filtration treatment, which was then directly used for another adsorption-desorption cycle. The adsorption-desorption cycle was repeated ten times. As shown in Figure 5f, after ten cycles of adsorption-desorption procedures, 98% removal of MB dye could be achieved, indicating that BJ160 possessed high stability, good recoverability, good regeneration characteristics. Therefore, PoBJ biochar might be used as a promising, sustainable, environmentally friendly, and efficient adsorbent for cationic dyes.

### 2.8. Adsorption of MB in Actual Water Samples

In order to investigate the adsorption of MB dye by BJ biochar in actual water samples, 50 mg L^−1^ solutions of MB dye were prepared using deionized water, industrial sewage and lake water (Yudai River), respectively. Compared to the MB solutions prepared using deionized water, a decrease in adsorption capacity of BJ160 for MB in both industrial wastewater (27.61 mg g^−1^) and Yudai River (125.04 mg g^−1^) could be observed (Figure 5g). The presence of a large amount of metal ions (e.g., Zn^2+^, Pb^2+^, Fe^3+^, Ca^2+^, etc.) in the actual water sample, especially in industrial wastewater, competed with MB molecules for the effective adsorption sites on BJ160, causing a sharp decrease in its adsorption capacity. The industrial wastewater tested in this study was provided by a local metallurgical plant, which contains inorganic metal ions such as Zn^2+^ and Pb^2+^. In addition, the industrial wastewater was acidic (pH = 3.73), which hindered the electrostatic interactions between BJ160 and MB due to the competitive adsorption effects of H^+^.

### 2.9. Adsorption Mechanisms

**Figure 6:**
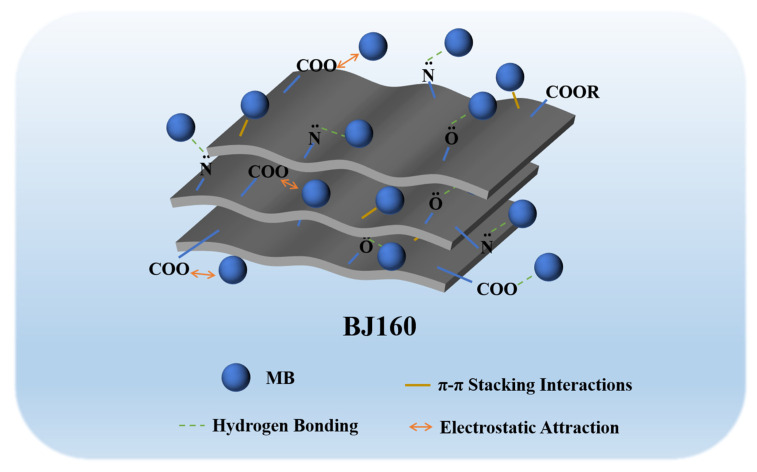
A proposed mechanism for MB adsorption by BJ160.

The structural properties, surface functional groups and charge density of an adsorbent dominated its adsorption capacity [50]. The adsorption of organic dyes by various biochar commonly occurs through multiple interactions including hydrogen bonding, ion exchange, electrostatic forces and π-π stacking interactions [40]. The characterization of the adsorbent pre- and post-adsorption could provide important information that allows us to reveal the possible adsorption mechanism. From FT-IR analyses, the successfully adsorption of MB onto BJ160 could be found, and the hydrogen bonding as well as the π-π stacking interactions could be confirmed. From zeta potential analysis, it can be concluded that BJ160 was negatively charged. The relatively lower pyrolysis temperature (160 °C) was far below 300 °C, allowing the good preservation of a large number of O-, N- and S-containing functional groups, which could interact with positively charged MB molecules by electrostatic attractions [51]. Meanwhile, the calculated Δ*H*° value (−29.42 kJ mol^−1^) fell within the range 1–40 kJ mol^−1^, again confirming the physical adsorption via electrostatic attraction which occurred between the adsorbent and the dye molecules at all temperatures tested [43]. In addition, cationic dyes were readily adsorbed by BJ160 while no adsorption to anionic dyes could be observed. Therefore, we could propose that the electrostatic attraction was the principal mechanism by which MB was adsorbed (Figure 6).

### 2.10. A Comparison with Other Adsorbents

The maximum adsorption capacity of BJ160 for MB was compared with those of several previously reported biochar (Table 1). It is evident that PoBJ biochar was prepared under relatively mild, low-emission and environmentally friendly conditions. The adsorption capacity of BJ160 for MB reached 160.11 mg g^−1^ at an initial MB concentration of 50 mg L^−1^ at room temperature, which was higher than most reported adsorbents [52]. In addition, BJ160 exhibited relatively superior adsorption selectivity toward cationic dyes rather than anionic dyes, which could be used to selectively adsorb/separate cationic dye from aqueous solutions.

## 3. Materials and Methods

### 3.1. Chemicals

The BJ was purchased from a vegetable market in Changsha (Hunan Province, China). Methylene blue (MB, 98.5 wt.%) was obtained from Beijing Chemical Factory. Alizarin Yellow R (AYR, 90 wt.%) was purchased from Hunan Xiangzhong Chemical Co. (Yueyang, China). Neutral Red (NR, 98 wt.%) was purchased from Tianjin Fuchen Chemical Reagent Co. (Tianjin, China). Azure I (AI) was collected from Shanghai Xin Sheng Test Chemical Technology Co. (Shanghai, China). Brilliant Green (BG), Calcein-Safranine (CS) and Rhodamine B (RB) were bought from National Pharmaceutical Base Chemical Reagent Co. (Shanghai, China). Hydrochloric acid (HCl, 37 wt.%) was obtained from Chengdu Kolon Chemical Co. (Chengdu, China). Sodium hydroxide (NaOH, 96 wt.%) was obtained from Tianjin Hengxing Chemical Preparation Co. (Tianjin, China). All other chemicals used in this study were of analytical grade and used without further purification.

### 3.2. Preparation of PoBJ-Based Biochar

The PoBJ stripped from BJ was naturally dried under room temperature to remove most of water, then further dried in an oven at 150 °C for 30 min. Afterwards, the PoBJ was cut into sheets and dried at 80 °C for 6 h. The dried PoBJ was ground in an agate mortar for 30 min to obtain powders. The ground PoBJ powders were placed in a porcelain boat, wrapped in tin foil and placed in a vacuum tube furnace (Model: SGM-6810A; Luoyang Sigma Instrument Manufacturing Co., Ltd.; Luoyang, China) for pyrolysis at different temperatures (160 °C, 200 °C, 240 °C, 280 °C, 320 °C and 360 °C). The pyrolysis of PoBJ powder was carried out in a horizontal tubular reactor, evacuated with an air pump until the pressure was reduced to −0.1 Mpa. The heating rate was 10 °C min^−1^ and the residence time was 60 min. The PoBJ-based biochar obtained at different temperatures were defined as BJ160, BJ200, BJ240, BJ280, BJ320 and BJ320, respectively. The PoBJ biochar was washed twice with ultrapure water and three times with anhydrous ethanol, then dried in an oven to a constant weight and finally transferred to a desiccator for future use.

### 3.3. Characterization of the Samples

The PoBJ biochar was characterized by SEM, FT-IR spectroscopy, XPS and zeta potential analysis. SEM (JEOL, JSM-6360LV; Tokyo, Japan) was used to observe the surface morphology of PoBJ biochar. XPS was applied to observe the functional groups and the compositions (including carbon (C), nitrogen (N), oxygen (O) and sulfur (S)) of the biochar. FT-IR spectrophotometer (model: Shimadzu IR Prestige 21; Tokyo, Japan) with a resolution of 4 cm^−1^ in the wavenumber range 4000–400 cm^−1^ was used to observe the functional groups and the changes of biochar before and after adsorption. Zeta potential analysis was carried out to elucidate the charge changes of biochar under various pH values.

### 3.4. Batch Adsorption Studies

The adsorption performances of PoBJ biochar for cationic, anionic and neutral dyes were investigated, and the differences in the adsorption capacities for these dyes were compared and discussed. The adsorption conditions were optimized and the adsorption kinetics, isotherms and thermodynamics were investigated by using MB as a model adsorbate. Adsorption experiments were carried out by adding 5.0 mg of BC to a conical flask (100 mL) containing 20.0 mL of dye solution with desired concentration. The concentration of the dye solution before and after adsorption were determined by a 752 UV-Vis spectrophotometer (Shanghai Jinghua Technology Instruments Co., Ltd.; Shanghai, China) at a specific wavelength (664 nm for MB, 625 nm for BG, 530 nm for CS, 648 nm for AI, 554 nm for RB, 370 nm for AYR and 542 nm for NR, respectively) at room temperature, and the dye concentrations were calculated by the calibration equations. The adsorption of MB onto PoBJ biochar prepared at different temperatures was carried out at 25 °C for 120 min. The optimization of adsorption conditions (contact time, temperature, initial MB concentration, contact temperature and solution pH) of BJ160 toward MB were explored in detail. The contact time (2 min, 5 min, 10 min, 15 min, 30 min, 60 min, 90 min, 120 min, 150 min) on BJ160 (5.0 mg) in 20.0 mL of MB (50 mg L^−1^) at 25 °C was studied. The effect of initial MB concentration (10 mg L^−1^, 20 mg L^−1^, 30 mg L^−1^, 40 mg L^−1^, 50 mg L^−1^) and temperature (25 °C, 30 °C, 35 °C, 40 °C, 45 °C) on the adsorption capacity of BJ160 to MB were investigated (*t* = 60 min, 5.0 mg of BJ160, 20.0 mL of MB). The effect of solution pH (2.24, 3.19, 4.24, 5.23, 6.23, 7.33, 7.22, 9.24) on the adsorption of MB (20.0 mL, 50.0 mg L^−1^) by BJ160 was carried out at 25 °C for 60 min. Aqueous hydrochloric acid (HCl, 0.1 mol L^−1^) and sodium hydroxide (NaOH, 0.1 mol L^−1^) solutions were used to adjust the solution pH values. For regeneration experiments, 50.0 mg of BJ160 was added to 20.0 mL of MB (50.0 mg L^−1^) for 60 min under shaking conditions. Desorption of MB from BJ160 was performed with 40.0 mL of hydrochloric acid (0.1 mol L^−1^).

The equilibrium adsorption capacity (*q_e_*, mg g^−1^) and removal rate (*R*, %) of PoBJ biochar for various adsorbents can be calculated by the following equations:

(1) $$q_{e}=\frac{(C_{0}-C_{e})\cdot V}{m}$$

(2) $$q_{t}=\frac{(C_{0}-C_{t})\cdot V}{m}$$

(3) $$R=\frac{C_{0}-C_{e}}{C_{0}}\times 100\%$$

where *C*_0_ (mg L ^−1^), *q_e_* (mg g^−1^), *C_e_* (mg L ^−1^), *q_t_* (mg g^−1^), *C_t_* (mg L ^−1^), and *V* (L), *m* (g), and *R* (%) are the initial adsorbate concentration, the equilibrium adsorption capacity of BJ material, and the equilibrium adsorbate concentration, the adsorption amount of BJ material at contact time of *t*, the adsorbate concentration at contact time of *t*, the volume of adsorbate solution, the mass of BJ material and the removal rate at contact time of *t*, respectively.

## 4. Conclusions

A facile and environmentally friendly method was used to prepare PoBJ biochars as efficient, cost-saving adsorbents for the selective adsorption of cationic dyes. BJ160 was characterized by FT-IR spectroscopy, XPS and SEM, and zeta potential analysis. By characterizing BJ160 pre- and post-adsorption of MB, the possible adsorption mechanism was proposed. The electrostatic interaction was found to dominate the adsorption of MB onto BJ160. The adsorption properties of BJ160 toward MB dye were investigated by batch adsorption experiments, and the experimental results showed that the newly prepared adsorbent was an efficient adsorbent with high adsorption capacity and selectivity. In addition, the adsorption thermodynamics shows that the adsorption is an exothermic process. After ten adsorption-desorption cycles, BJ160 still exhibited a high removal rate toward MB, indicating it possessed high stability and good reusability. Therefore, the rationally designed and facilely prepared PoBJ biochar might have a broad potential for the treatment of dye-containing wastewater in the future.

## Figures and Tables

**Table 1:** A comparison of adsorption capacities of BJ160 toward MB with other previously reported carbon-based adsorbents under optimal experimental conditions.

Adsorbent	Activated Method/Activator	T (K)	Adsorbent Dosage (g L^−1^)	C_MB_ (mg/L)	*q*_max_ (mg/g)	Ref.
Heavy bio-oil	Furnace (800 °C, N_2_)	303	2.0	1000	411	[25]
Eggshell membranes	KOH and HNO_3_/Furnace (200 °C, N_2_)	298	1.25	100	110.38	[26]
Rice straw	Toxicity characteristic leaching procedure/Furnace (500 °C, N_2_)	298	3.0	1000	51.34	[53]
Date seed	Muffle furnace (700 °C)	298	20.0	1279.4	42.57	[54]
Reed	Tubular furnace (500 °C, N_2_)	298	1.0	50	77.35	[55]
water hyacinth	Tube furnace (300 °C, N_2_)	303	1.0	200	395	[56]
BJ160	Vacuum tube furnace (160 °C)	293	0.25	60	185.63	This work

## Data Availability

Data sharing is not applicable.

## Supplementary Materials

The following supporting information can be downloaded at: https://www.mdpi.com/article/10.3390/molecules28083353/s1, Figure S1: (a) Fitted adsorption kinetic curves by nonlinear pseudo-first-order models; (b) Fitted adsorption kinetic curves by nonlinear pseudo-second-order models; Figure S2: (a) The experimental data of BJ160 toward MB and the fitting curves of Langmuir isotherm model; (b) The experimental data of BJ160 toward MB and the fitting curves of Freundlich isotherm model; Figure S3: Experimental data and the fitted curve of InK° eq versus 1/T calculated from van’t Hoff plots of adsorption of MB onto BJ160; Table S1: Adsorption kinetic parameters for the adsorption of MB onto BJ160; Table S2: Adsorption isothermal parameters for the adsorption of MB onto BJ160; Table S3: Thermodynamic parameters of the adsorption of MB onto BJ160.
