# Supplementary material for: Efficient and Selective Removal of Organic Cationic Dyes by Peel of Brassica juncea Coss. var. gemmifera Lee et Lin-Based Biochar
Source: Molecules. 2023 Apr 11;28(8):3353. doi: 10.3390/molecules28083353 (PMC10143088; doi:10.3390/molecules28083353)
Supplement: Supplementary file 1 [file molecules-28-03353-s001.zip › molecules-2318645-supplementary.pdf]

Efficient and selective removal of organic cationic dyes by peel of Brassica juncea Coss. var. gemmifera Lee et Lin-based biochar

Tao-Tao Shi[1], Xin-Yu Jiang[1], Jin-Gang Yu[1,*]

[1]*College of Chemistry and Chemical Engineering, Central South University, Changsha, Hunan 410083, China*

1. Adsorption kinetics

Two couples of kinetic models were used to fit the experimental data of contact time on MB adsorbed onto the biochar derived from PoBJ. The first models, nonlinear pseudo-first-order models, could be expressed as follows:

$$q_t = q_e(1 - e^{-k_1 t}) \quad (1)$$

The second models, nonlinear pseudo-second-order models, could be expressed as follows:

$$q_t = \frac{k_2 q_e^2 t}{1 + k_2 q_e t} \quad (2)$$

where t (min) is the contact time; q_e (mg/g) and q_t (mg/g) are the amounts of MB adsorbed at equilibrium and at time t, respectively; k_1 (min^{-1}) and k_2 (g/(mg·min)) are the specific adsorption rate constants of the pseudo first-order model and the pseudo second order models, respectively.

To whom correspondence should be addressed.

[*] JG Yu, E-mail: yujg@csu.edu.cn; Tel/Fax: +86-731-88879616.

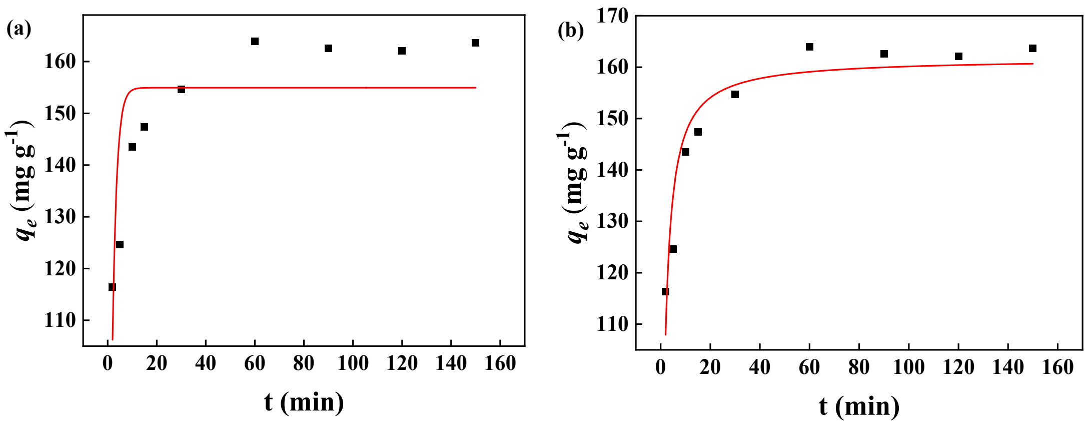

Figure S1 (a)Fitted adsorption kinetic curves by nonlinear pseudo-first-order models; (b) Fitted adsorption kinetic curves by nonlinear pseudo-second-order models.

Table S1 Adsorption kinetic parameters for the adsorption of MB onto BJ160.

Fitting parameters	Non-linear pseudo-first-order model	Non-linear pseudo-second-order model
R^2	0.537	0.885
k_1 (min^{-1})	0.579	-
k_2 (g mg^{-1}min^{-1})	-	0.0062
q_e (mg/g)	154.9	161.7

2. Adsorption isotherms and thermodynamics

Two isothermal models were used to fit the experimental data of MB adsorbed on BJ160.

Langmuir model:

$$q_e = \frac{q_m k_L C_e}{1 + k_L C_e} \tag{3}$$

where C_e (mg/L) is the equilibrium concentration; q_e (mg/g) is the amount of adsorbed species per specified amount of adsorbent; K_L is the Langmuir equilibrium constant and q_m (mg/g) is the amount of adsorbate required to form an adsorbed monolayer.

Freundlich model:

$$q_e = k_F C_e^{\frac{1}{n}} \tag{4}$$

where C_e (mg/L) is the equilibrium concentration of adsorbent; q_e (mg/g) is the amount of adsorbent per unit mass; K_F and n are two Freundlich constants: n represents the relative advantage of adsorption process; K_F is the affinity constant which is related to the adsorption capacity of the adsorbent and can also be defined as adsorption or distribution coefficient, indicating the amount of dye adsorbed on BJ160 at equilibrium concentration.

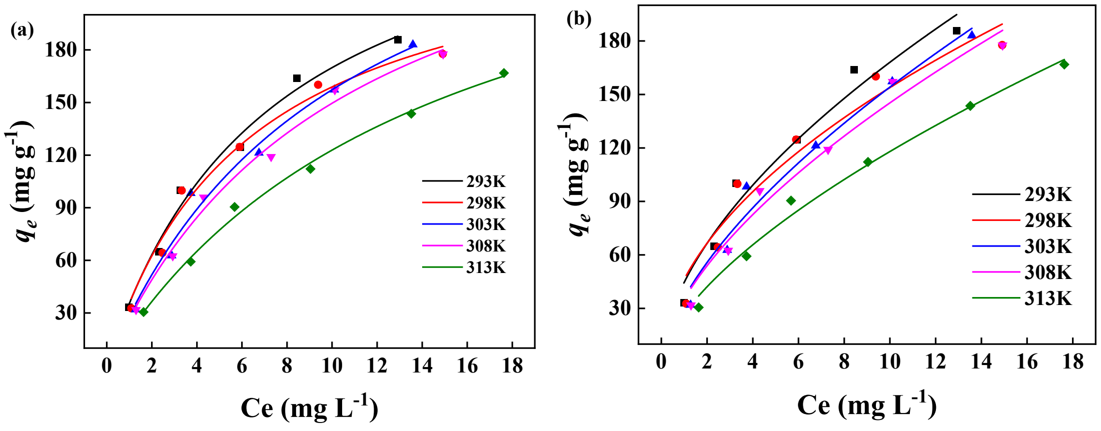

Figure S2 (a)The experimental data of BJ160 toward MB and the fitting curves of Langmuir isotherm model; (b) The experimental data of BJ160 toward MB and the fitting curves of Freundlich isotherm model.

Table S2 Adsorption isothermal parameters for the adsorption of MB onto BJ160.

Model	Fitting parameters	293K	298K	303K	308K	313K
Langmuir	R^2	0.984	0.979	0.979	0.987	0.995
	k_L	0.14	0.16	0.096	0.095	0.069
	q_m (mg/g)	293.9	258.2	320.1	307.5	300.1
Freundlich	R^2	0.960	0.931	0.967	0.967	0.987
	k_F	44.61	46.37	35.99	35.17	27.05
	n	1.74	1.92	1.58	1.62	1.56

Thermodynamic parameters including enthalpy ($\triangle H°$, kJ/mol), entropy ($\triangle S°$, J/(K·mol)) and Gibbs free energy ($\triangle G°$, kJ/mol) of the adsorption process can be calculated by **Eq. (9-11).**

$$K_{eq}^{\circ} = \frac{K_L}{\gamma_{Adsorbate}} \times C^{\circ} \tag{5}$$

$$\ln K_{eq}^{\circ} = \frac{-\Delta G^{\circ}}{RT} = \frac{\Delta S^{\circ}}{R} - \frac{\Delta H^{\circ}}{RT} \tag{6}$$

$$\Delta G^{\circ} = \Delta H^{\circ} - T\Delta S^{\circ} \tag{7}$$

where K_{eq}° is the thermodynamic equilibrium constant; K_L is the Langmuir equilibrium constant; C° =1mol/L; $\gamma_{\mathrm{Adsorbate}}$ is the activity coefficient of the adsorbent; T (K) is the absolute temperature and R (8.314 J/(mol·K)) is the ideal gas constant.

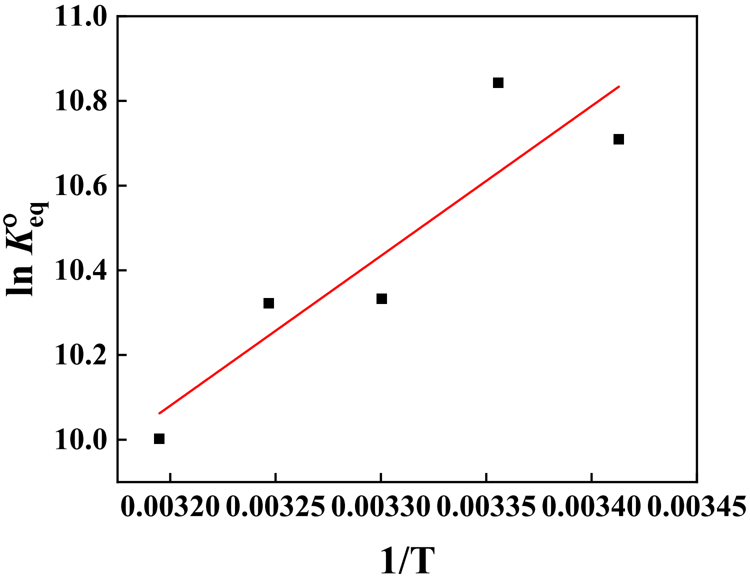

Figure S3 Experimental data and the fitted curve of InK°_{eq} versus 1/T calculated from van't Hoff plots of adsorption of MB onto BJ160.

Table S3 Thermodynamic parameters of the adsorption of MB onto BJ160.

T **(K)**	**△*G*° (kJ/mol)**	**△*H*° (kJ/mol)**	**△*S*° (J/(K·mol))**
293K	-26.39		
298K	-26.34		
303K	-26.29	-29.42	-10.33
308K	-26.24		
313K	-26.18		
